# Comparative Analysis of Vonoprazan- and Dexlansoprazole-Based Therapies for Helicobacter pylori Eradication: A Single-Center Retrospective Observational Study

**DOI:** 10.7759/cureus.117041

**Published:** 2026-09-27

**Authors:** VG Mohan Prasad, Vamsi Murthy K, Madhura Prasad Suman, Mithra Prasad, Jackson Keefer Jerry

**Affiliations:** 1 Gastroenterology, VGM Hospital, Coimbatore, IND

**Keywords:** dexlansoprazole, helicobacter pylori, potassium-competitive acid blocker, proton pump inhibitors, vonoprazan

## Abstract

Background and aim

Vonoprazan, a potassium-competitive acid blocker (PCAB), has emerged as an alternative to proton pump inhibitors (PPIs) for *Helicobacter pylori* eradication. This study assessed the comparative effectiveness of vonoprazan- versus dexlansoprazole-based regimens in a real-world clinical setting.

Methods

We retrospectively analyzed 146 patients (73 per group) receiving vonoprazan- or dexlansoprazole-based therapy. Eradication success rates were compared using both frequency-based and Bayesian methods, with additional risk estimates and time-to-event analyses.

Results

The vonoprazan group had a higher eradication rate (93.15%, 95% CI: 84.95, 97.04) compared with dexlansoprazole (83.56%, 95% CI: 73.43, 90.34); this difference did not reach statistical significance at the conventional 0.05 level (χ² = 3.262, p = 0.07). Risk ratio (1.11, 90% CI: 1.01, 1.23) and odds ratio (2.68, 90% CI: 1.06, 6.73) estimates using exploratory 90% CIs excluded the null at this wider, non-confirmatory threshold, favoring vonoprazan, though the corresponding 95% CIs remained inconclusive. Bayesian analysis yielded a posterior risk difference of 9.3% (95% credible interval: -1.1, 20.1) and a 96.2% posterior probability that vonoprazan was more effective, though the credible interval still included values close to and below zero. Kaplan-Meier analysis showed a shorter median time to documented confirmation of eradication with vonoprazan (97 days) than with dexlansoprazole (114 days; log-rank p = 0.02), which reflects the timing of documented testing rather than a direct measure of biological eradication speed.

Conclusions

Vonoprazan was associated with a numerically higher eradication rate and earlier documented confirmation of eradication compared with dexlansoprazole. While the primary comparison did not reach statistical significance at the 95% level, several exploratory analyses, including the 90% CIs and the Bayesian posterior probability, were directionally consistent with a possible advantage for vonoprazan; these findings are hypothesis-generating rather than confirmatory. Larger, multicenter, prospective studies are warranted to confirm these results.

## Introduction

*Helicobacter pylori* infects nearly half of the global population, with a prevalence of 43.7% in adults and 34.4% in children/adolescents from 2015 to 2022 [1]. However, prevalence is significantly influenced by geographic location, sanitation, and living conditions [2]. *H. pylori* invasion in the stomach can trigger chronic gastritis that may progress to peptic ulcer, gastric cancer, and gastric mucosa-associated lymphoma [2,3]. Standard regimens to treat the infection employ proton pump inhibitors (PPIs) in combination with antibiotics [4,5]. PPIs such as omeprazole, pantoprazole, and dexlansoprazole manage acid-related gastrointestinal disorders by irreversibly inhibiting the H⁺/K⁺-ATPase enzyme system in gastric parietal cells [6]. However, there are growing concerns regarding PPI-associated risks, including bone fractures, micronutrient deficiencies, renal impairment, and dementia [7].

Despite their widespread use, PPIs have notable limitations: delayed onset, inconsistent acid suppression due to CYP2C19 metabolism, risk of tachyphylaxis with prolonged use, and acid lability requiring enteric coating [8-10]. In contrast, potassium-competitive acid blockers (PCABs) like vonoprazan offer rapid, potent, and consistent acid suppression. They are acid-stable and do not require gastric activation [11-13]. Thus, PCABs are a promising alternative when PPI therapy is inadequate or stronger acid suppression is required. Vonoprazan has demonstrated strong potential for effective *H. pylori* eradication.

With an estimated 80%-90% *H. pylori* seroprevalence and rising antibiotic resistance in India, optimizing acid suppression strategies is critical [14,15]. CYP2C19 polymorphisms, common in Asian populations, further contribute to variable PPI efficacy [16,17]. In this context, the consistent and potent acid suppression by PCABs like vonoprazan offers a potential clinical advantage. Therefore, evaluating vonoprazan’s real-world effectiveness in the Indian population is clinically relevant.

Comparative data on the effectiveness of vonoprazan and PPIs such as dexlansoprazole are limited in the Indian context. This study aimed to compare the real-world effectiveness of vonoprazan- and dexlansoprazole-based regimens for *H. pylori* eradication in a representative subset of the Indian population. Dexlansoprazole was selected as the comparator PPI because of its dual delayed-release formulation, which provides prolonged, consistent acid suppression compared with conventional PPIs [18].

## Materials and methods

Study design and setting

This was a single-center, retrospective, observational cohort study conducted at VGM Hospital, Institute of Gastroenterology, Coimbatore, Tamil Nadu, India - a tertiary referral center for gastrointestinal disease in South India. Data were extracted from hospital electronic medical records and prescription logs of patients diagnosed with *H. pylori* infection between 2021 and 2025. The protocol was approved by the Institutional Ethics Committee, VGM Hospital, Institute of Gastroenterology (approval letter dated 27 November 2024). The study was conducted in accordance with the Declaration of Helsinki.

Participants

Adults aged 18 years or older with dyspeptic symptoms and a confirmed diagnosis of *H. pylori* infection, established either by endoscopic biopsy-based testing or by 13C urea breath test where endoscopy was unavailable or declined, were eligible. The predominant biopsy-based diagnostic method was the rapid urease test performed on endoscopic biopsy specimens; a proportion of patients also had histological confirmation. Culture-based diagnosis was not routinely performed. Of 146 included patients, 90 (61.6%) were diagnosed by endoscopic biopsy-based methods and 56 (38.4%) by 13C urea breath test.

Exclusion criteria were previous *H. pylori *eradication therapy of any kind, antibiotic or bismuth use within the four weeks preceding the study regimen, allergy or intolerance to any study medication (except quinolones), pregnancy or lactation, gastric malignancy, prior gastric surgery, severe hepatic or renal impairment, incomplete clinical records, and inability to comply with follow-up.

Rationale for the antibiotic backbone

The choice of amoxicillin plus levofloxacin plus bismuth (rather than the conventional tetracycline plus metronidazole backbone) was driven by well-documented Indian resistance patterns accumulated over three decades. Metronidazole resistance in Indian *H. pylori* isolates approaches 78% and clarithromycin resistance exceeds 30% in several regions, making metronidazole- and clarithromycin-containing regimens clinically unreliable in routine practice, where pre-treatment susceptibility testing is not available [19,20]. Levofloxacin has therefore emerged as the preferred alternative in Indian bismuth-based regimens. The addition of bismuth to levofloxacin-based therapy has been shown in meta-analyses to increase eradication rates by approximately 8%-12% relative to levofloxacin triple therapy, particularly after prior clarithromycin failure [21]. Amoxicillin was retained because primary amoxicillin resistance in India remains comparatively low. This regimen is consistent with the Maastricht VI/Florence consensus, which endorses bismuth-containing levofloxacin-based regimens as first-line therapy in settings of high or unknown clarithromycin resistance [21]. All 146 included patients received this regimen as first-line eradication therapy; patients with any previous *H. pylori* eradication attempt were excluded per the eligibility criteria.

Treatment regimen 

Both regimens used the same 14-day antibiotic backbone: amoxicillin 500 mg three times daily, levofloxacin 500 mg once daily, and bismuth subcitrate 240 mg twice daily, with *Limosilactobacillus reuteri* DSM 17938 as an adjunct probiotic. The acid-suppressant component was either vonoprazan 20 mg twice daily or dexlansoprazole 60 mg once daily. Treatment allocation was determined by the prescribing physician's preference at the time of endoscopy and prescription. In two patients (1.4%: one in the dexlansoprazole arm and one in the vonoprazan arm) with known quinolone allergy or intolerance, levofloxacin was replaced with doxycycline 100 mg twice daily; amoxicillin and bismuth were unchanged. A pre-specified sensitivity analysis restricted to the 144 patients receiving the standard antibiotic backbone is reported.

Outcome ascertainment

The primary outcome was *H. pylori* eradication, assessed 6-10 weeks after completion of therapy by 13C urea breath test (Heliprobe/PyloPLUS™ system) or repeat upper gastrointestinal endoscopy with biopsy where clinically indicated. All acid-suppressant therapy, antibiotics, and bismuth were discontinued for at least four weeks prior to the confirmatory test, exceeding the international consensus recommendation of ≥2 weeks for PPIs/PCABs and ≥4 weeks for antibiotics and bismuth, and thereby minimizing the risk of false-negative results. This washout was applied uniformly to both arms.

To assess treatment effect, the eradication rates were compared between the vonoprazan and dexlansoprazole groups. Risk estimates included risk ratio, odds ratio, absolute risk difference, and number-needed-to-treat, with corresponding 95% confidence intervals (CIs) as the primary measure of precision. Exploratory 90% CIs for the same estimates are also reported to characterize potentially meaningful trends where the 95% intervals were inconclusive; these exploratory estimates were not used for confirmatory inference. As an exploratory analysis of this same primary outcome, we also used a Bayesian method to estimate and compare the eradication rates of vonoprazan and dexlansoprazole. Given binary outcomes, posterior distributions were modeled using Beta distributions, assuming non-informative Beta(1,1) priors. Posterior parameters were defined as Beta(1 + successes, 1 + failures), yielding Beta(69, 6) for vonoprazan and Beta(62, 13) for dexlansoprazole. Using 100,000 Monte Carlo samples drawn from these posteriors, we estimated the risk difference (vonoprazan minus dexlansoprazole), the 95% credible interval (CrI), and the probability that vonoprazan is superior. As a further exploratory analysis, Kaplan-Meier analysis was employed to assess the time to documented confirmation of eradication rather than as a direct measure of underlying biological eradication speed. For this time-to-event analysis, time to documented confirmation of eradication was defined as the number of days from treatment completion to the date of the first documented negative urea breath test for each patient. Because confirmatory testing occurred at variable real-world follow-up intervals rather than at a single fixed time point, the duration varied across patients and served as the time-to-event endpoint, reported separately from the binary primary outcome. Patients without a documented negative breath test were censored at the date of their last recorded follow-up.

Adherence assessment

Treatment adherence was reviewed at the follow-up visit by clinician interview and by review of pharmacy dispensing records. All 146 included patients were considered adherent to their prescribed regimen based on this composite clinical assessment; no patient was excluded from the analytic cohort for documented non-adherence.

Statistical analysis 

Descriptive statistics summarize baseline characteristics, including age and gender. Categorical variables were reported as counts and percentages, and continuous variables as means ± standard deviations (SDs). Success rates (95% CI) were compared between treatment arms using the Chi-square test; p < 0.05 indicated statistical significance. Comorbidity comparisons reported in the Results section were instead assessed using Fisher's exact test, given the low frequency of several categories and the resulting small expected cell counts. The 95% CI was the primary basis for inference for all risk estimates (risk ratio, odds ratio, and absolute risk difference). Because several 95% intervals were inconclusive, exploratory 90% CIs for the same estimates are also reported to characterize potentially meaningful trends; findings based on the 90% CI are interpreted as hypothesis-generating only and do not alter the primary conclusions, which rest on the 95% CI results. Bayesian analysis quantified the posterior probability of vonoprazan's superiority over dexlansoprazole using a uniform (non-informative) prior. Posterior distributions for success rates and the risk difference were generated using Beta distributions, with 95% CrI reported. Kaplan-Meier time-to-event analysis utilized the log-rank test to compare survival curves. Standardized effect size was calculated as Cohen's h. Analyses were performed in RStudio version 2024.12.1+563 (Posit Software, PBC, Boston, MA, USA).

## Results

Study population

Between 2021 and 2025, 146 consecutive adults with dyspeptic symptoms and confirmed *H. pylori* infection were included in the analysis, with 73 in each treatment arm (Figure 1). All 146 patients received at least one dose of allocated therapy and underwent the confirmatory eradication assessment.

**Figure 1:**
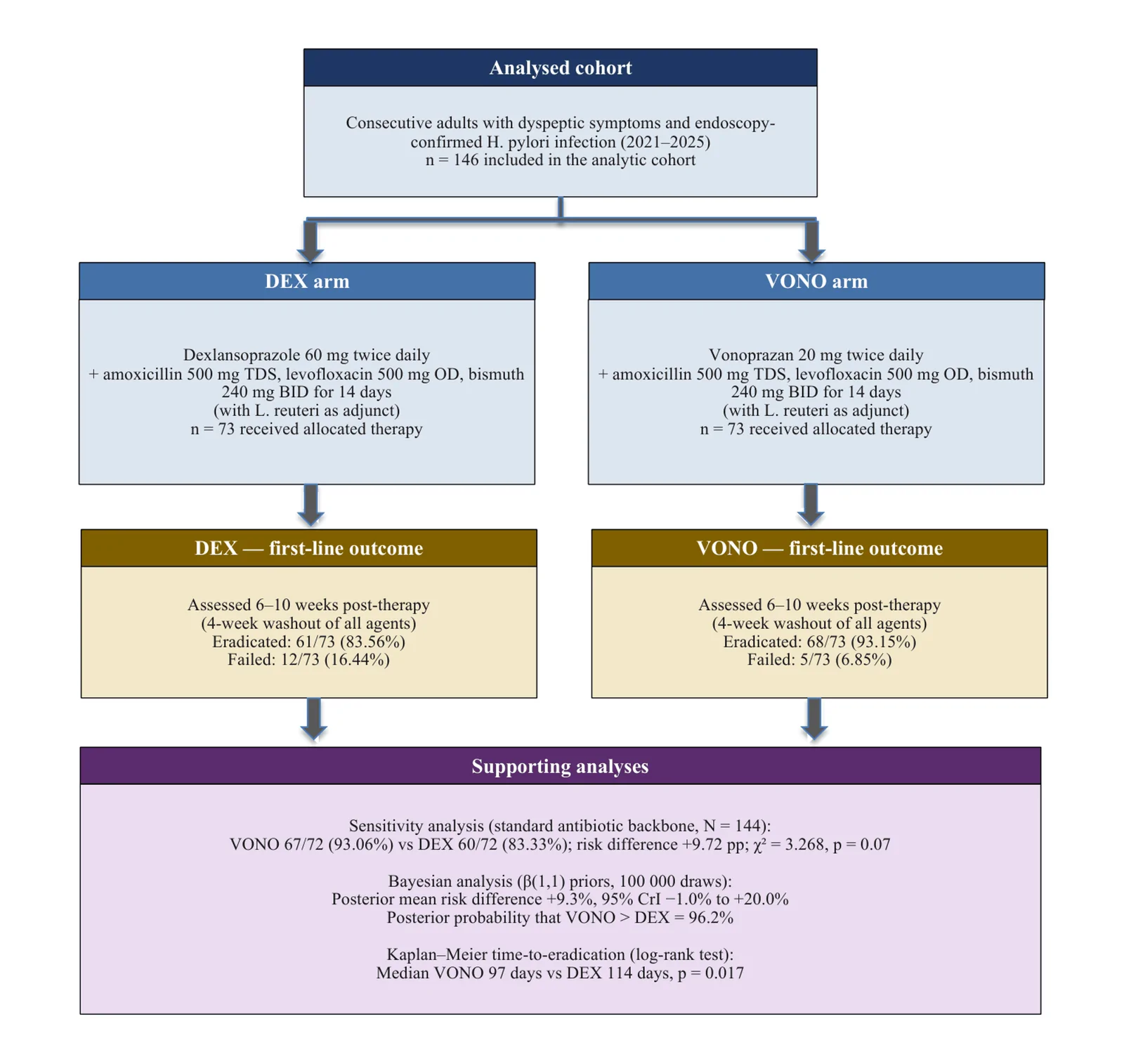
Study flow diagram Flow of the 146 adults included in the analytic cohort (73 per treatment arm), receipt of allocated therapy, and assessment of the primary eradication endpoint 6-10 weeks after therapy, following a four-week washout of all agents. The primary comparison did not reach conventional significance (χ² = 3.262, p = 0.07), but several exploratory analyses were directionally consistent with a possible advantage for vonoprazan, though none were confirmatory. DEX, dexlansoprazole-based bismuth quadruple therapy; VONO, vonoprazan-based bismuth quadruple therapy; *H. pylori*, *Helicobacter pylori*

Baseline characteristics

Baseline characteristics were balanced between the two arms (Table 1). Sex distribution was comparable (vonoprazan 45/73 (61.64%) male; dexlansoprazole 43/73 (58.90%) male; p = 0.87). The mean age was 48.9 years (SD 15.1) in the vonoprazan arm and 46.1 years (SD 16.3) in the dexlansoprazole arm (p = 0.30). Age stratification did not differ significantly between arms (p = 0.17). The diagnostic method was similarly distributed (biopsy-based diagnosis in 46 vonoprazan vs 44 dexlansoprazole; 13C urea breath test in 27 VONO (vonoprazan-based bismuth quadruple therapy) vs 29 DEX (dexlansoprazole-based bismuth quadruple therapy); p = 0.87). The antibiotic backbone was also balanced (standard amoxicillin plus levofloxacin in 72 vonoprazan vs 72 dexlansoprazole; doxycycline substitution in one VONO vs one DEX for quinolone allergy or intolerance; p = 1.00). Comorbidity burden and duodenal ulcer status were also comparable between arms across all 11 categories assessed (Table 2; all p ≥ 0.12, Fisher's exact test).

**Table 1:** Baseline characteristics of the study population (N = 146) Values are mean ± SD or n (%). Sex and diagnostic method compared by χ² test; age (mean) compared by independent-samples t-test; age group compared by χ² test; antibiotic backbone compared by Fisher's exact test, given the low expected cell counts. Rapid urease test (RUT) performed on endoscopic biopsy specimens.

Characteristic	Vonoprazan (n = 73)	Dexlansoprazole (n = 73)	p-value
Demographics			
Sex, n (%)			0.87
Male	45 (61.64)	43 (58.90)	-
Female	28 (38.36)	30 (41.10)	
Age, years, mean ± SD	48.9 ± 15.1	46.1 ± 16.3	0.30
Age range, years	19 - 87	18 - 77	-
Age group, n (%)			0.17
18 - 20	2 (2.74)	5 (6.85)	-
21 - 30	6 (8.22)	8 (10.96)	
31 - 40	13 (17.81)	18 (24.66)	
41 - 50	22 (30.14)	9 (12.33)	
51 - 60	15 (20.55)	17 (23.29)	
61 - 70	9 (12.33)	12 (16.44)	
71 - 80	4 (5.48)	4 (5.48)	
81 - 90	2 (2.74)	0 (0.00)	
Diagnostic Method, n (%)			0.87
Endoscopic biopsy (predominantly RUT)	46 (63.01)	44 (60.27)	-
13C urea breath test	27 (36.99)	29 (39.73)	
Antibiotic Backbone, n (%)			1.000 (Fisher's exact)
Amoxicillin + Levofloxacin	72 (98.63)	72 (98.63)	-
Doxycycline (substituted for quinolone allergy)	1 (1.37)	1 (1.37)	

**Table 2:** Primary comparison of H. pylori eradication and estimated risk measures with 95% and 90% confidence intervals Risk ratio was computed by the log method, odds ratio by the Woolf method, and risk difference by the Wald method. The primary comparison used Pearson's χ² test without continuity correction; Fisher's exact test is provided for reference. The sensitivity analysis excluded two patients receiving doxycycline substitution. CI, confidence interval; pp, percentage points; RD, risk difference; VONO, vonoprazan-based bismuth quadruple therapy; DEX, dexlansoprazole-based bismuth quadruple therapy; *H. pylori*, *Helicobacter pylori*

Metric	Point estimate	95% CI	90% CI
Eradication, VONO (n/N, %)	68/73 (93.15%)	84.95% - 97.04%	-
Eradication, DEX (n/N, %)	61/73 (83.56%)	73.43% - 90.34%	-
χ² (df = 1)/p-value	χ² = 3.262	-	p = 0.07
Risk ratio (VONO/DEX)	1.115	(0.989, 1.256)	(1.009, 1.232)
Odds ratio (VONO/DEX)	2.675	(0.891, 8.030)	(1.064, 6.730)
Absolute risk difference (pp)	9.59	(-0.70, +19.88)	(+0.95, +18.22)
Number needed to treat	11	-	-
Fisher's exact test/p-value	-	-	p = 0.12
Sensitivity analysis (standard backbone, N = 144)	VONO 93.06% vs DEX 83.33%	RD +9.72 pp	p = 0.07

Primary outcome - risk-estimate analyses

VONO achieved *H. pylori* eradication in 68 of 73 patients (93.15%; 95% CI 84.95-97.04%), compared with 61 of 73 patients (83.56%; 95% CI 73.43-90.34%) receiving DEX (Table 3 and Figure 2). The absolute risk difference was +9.59 percentage points in favor of vonoprazan (95% CI -0.70 to +19.88), corresponding to a number needed to treat of 11. The risk ratio was 1.115 (95% CI 0.989-1.256) and the odds ratio was 2.675 (95% CI 0.891-8.030). This primary comparison did not reach conventional statistical significance (χ² = 3.262, p = 0.07); the direction and magnitude of the point estimates were consistent with a numerically higher eradication rate for vonoprazan, but statistical superiority was not established.

**Table 3:** Comorbidity categories and duodenal ulcer status by treatment group (N = 146) Values are n (%) unless otherwise stated. Comparisons between the vonoprazan and dexlansoprazole arms were made using Fisher's exact test, given the low frequency of several categories and the resulting small expected cell counts.

Comorbidity	Vonoprazan (n = 73)	Dexlansoprazole (n = 73)	p-value (Fisher's exact)
Any comorbidity	45 (61.6%)	41 (56.2%)	0.61
Type 2 diabetes mellitus	12 (16.4%)	13 (17.8%)	1.00
Hypertension	17 (23.3%)	14 (19.2%)	0.69
Hyperlipidemia	29 (39.7%)	26 (35.6%)	0.73
Obesity or overweight	9 (12.3%)	15 (20.5%)	0.26
Hypothyroidism	5 (6.8%)	4 (5.5%)	1.00
Ischemic or cardiovascular disease	0 (0.0%)	4 (5.5%)	0.12
Liver disease	4 (5.5%)	1 (1.4%)	0.37
Vitamin B12 deficiency	2 (2.7%)	4 (5.5%)	0.68
Anemia	2 (2.7%)	3 (4.1%)	1.00
Duodenal ulcer	6 (8.2%)	2 (2.7%)	0.28

**Figure 2:**
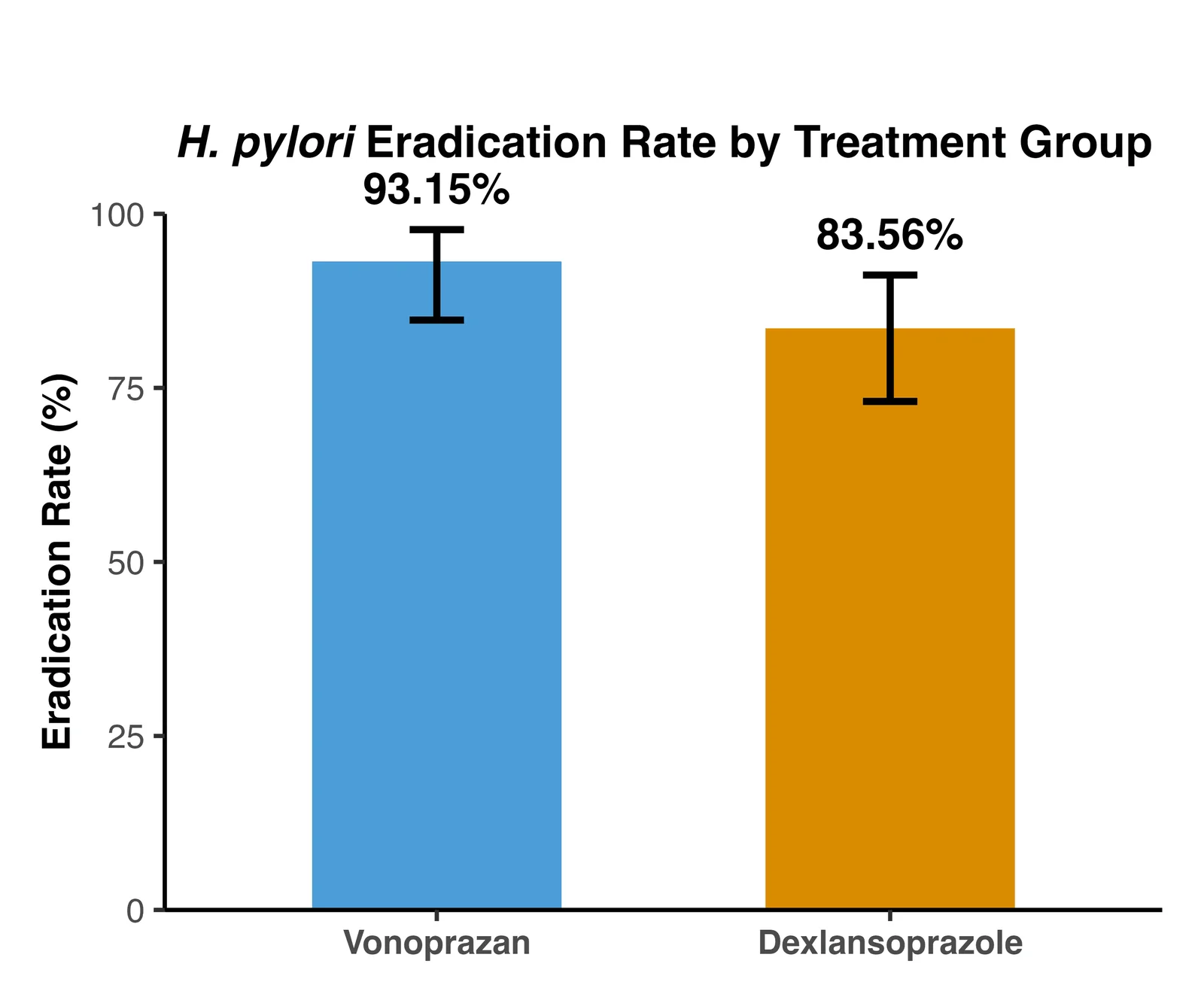
H. pylori eradication success rates by treatment group Bar graph comparing the eradication success rates of vonoprazan (n = 73) and dexlansoprazole-based (n = 73) regimens. Pearson’s chi-square test was used to calculate the statistical significance (χ² = 3.262, p = 0.07). Error bars represent 95% confidence intervals. H. pylori, Helicobacter pylori

Exploratory analyses using 90% CIs

The 95% CI for these risk estimates included the null, so the primary analysis remained statistically inconclusive for the risk ratio, odds ratio, and absolute risk difference. As an exploratory analysis, we also examined the same point estimates using the wider 90% CI (Table 3); under this exploratory threshold, the risk ratio was 1.11 (90% CI: 1.01, 1.23), the odds ratio was 2.68 (90% CI: 1.06, 6.73), and the absolute risk difference was 9.6% (90% CI: 0.95%, 18.2%). These exploratory findings are hypothesis-generating only and should not be read as establishing statistical superiority of vonoprazan; the conventional primary analysis based on the 95% CI did not reach significance for any of these measures.

Exploratory analysis - Bayesian estimation of treatment effect

To quantify the uncertainty in traditional frequentist comparisons, we performed an exploratory Bayesian analysis (Figure 3 and Appendix Tables 4-5). Using non-informative uniform priors (β(1,1)) and equal sample sizes (n = 73 per group), the posterior success rate was 93.3% (95% CrI: 84.9%, 97.0%) for vonoprazan and 83.6% (95% CrI: 73.3%, 90.3%) for dexlansoprazole (Figure 3A). The posterior risk difference was 9.3% (95% CrI: -1.1%, 20.1%), with a right-skewed distribution suggesting a potential absolute advantage with vonoprazan; however, because the 95% CrI includes both negative and positive values, this result remains statistically uncertain (Figure 3B). The posterior probability that the risk difference exceeds zero was 96.2%. Because the corresponding credible interval still includes no benefit and a small possible disadvantage, we present this posterior probability as an exploratory estimate of the direction of effect rather than as definitive evidence of vonoprazan's superiority.

**Figure 3:**
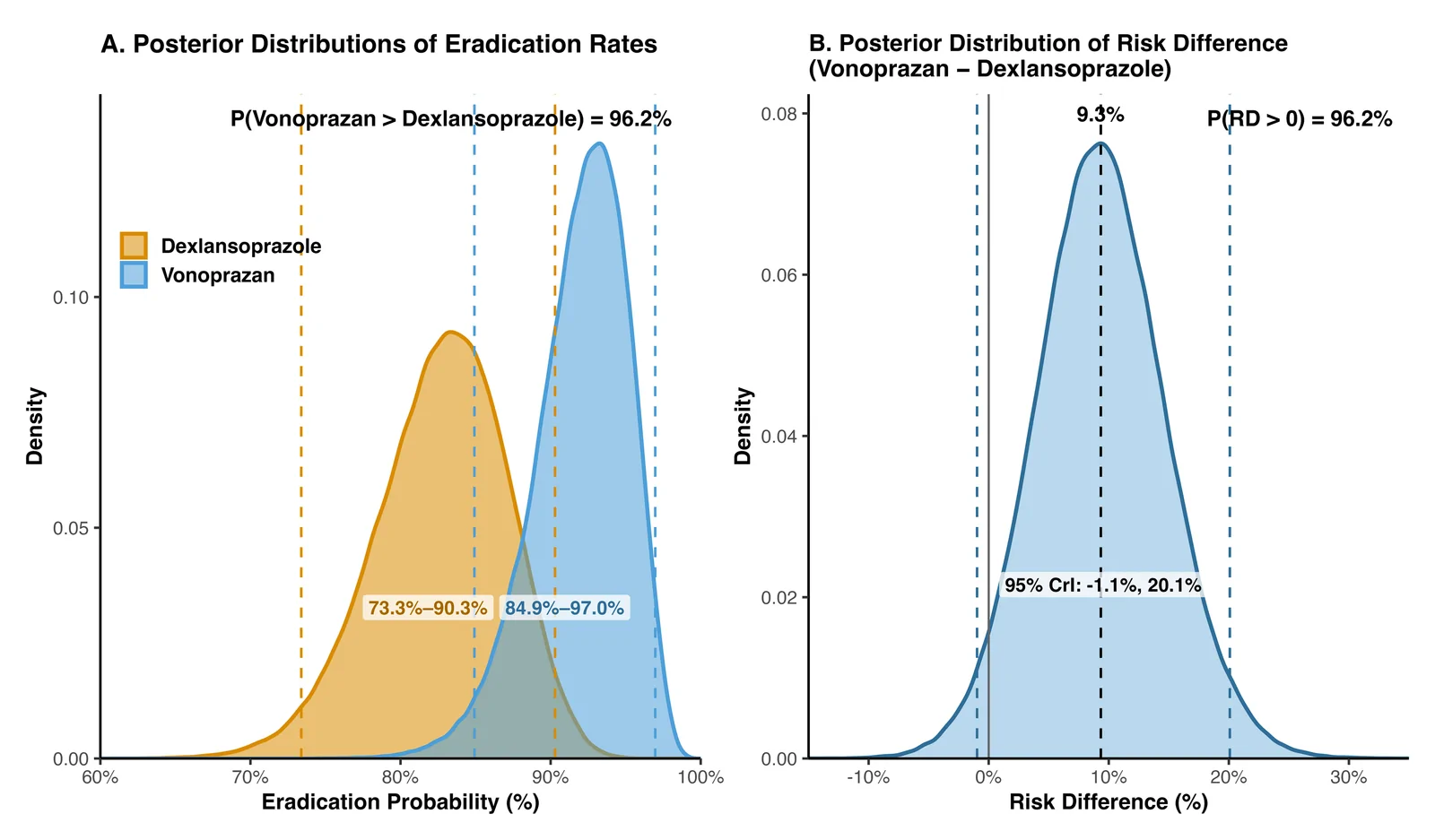
Bayesian posterior estimates of H. pylori eradication rates and risk difference by treatment group (A) Posterior mean eradication success rates for vonoprazan (n = 73) and dexlansoprazole (n = 73), modeled using a beta-binomial framework with non-informative uniform priors (β(1,1)); 95% CrI; dashed lines mark each group's 95% CrI. (B) Posterior distribution of the risk difference between vonoprazan and dexlansoprazole; 95% CrI. The distribution was right-skewed, with a posterior risk difference of 9.3% (95% CrI: -1.1%, 20.1%), suggesting a potential absolute advantage with vonoprazan; because the interval spans zero, this reflects statistical uncertainty rather than a definitive effect. *H. pylori, Helicobacter pylori*; CrI, credible interval

Exploratory analysis - time to documented confirmation of eradication

Next, we performed a Kaplan-Meier analysis of the time to documented confirmation of eradication between treatment groups, with each patient's duration calculated from treatment completion to the date of the first documented negative urea breath test (Figure 4). Because confirmatory testing occurred at variable, non-protocol-driven follow-up intervals rather than at a single fixed time point in this retrospective cohort, this analysis reflects the timing of documented confirmation rather than a direct measure of underlying biological eradication speed and is presented as exploratory and descriptive. The interval between treatment completion and confirmatory testing varied widely in both arms: median 67 days (IQR 61-96 days) in the vonoprazan group and median 80 days (IQR 64-185 days) in the dexlansoprazole group. The median time to documented confirmation was numerically shorter in the vonoprazan group (97 days, 95% CI: 85.85, 108.16) than in the dexlansoprazole group (114 days, 95% CI: 88.61, 139.39); the log-rank test comparing the two curves gave p = 0.02. Given the wide and uneven spread of testing intervals in both arms, particularly the dexlansoprazole group's interquartile range extending to 185 days, we cannot exclude that this difference partly reflects differential follow-up scheduling between arms rather than a true difference in biological eradication speed, and we do not interpret this finding as evidence that vonoprazan produces faster biological eradication. The standardized effect size for the primary eradication-rate comparison (Cohen's h) was 0.31, consistent with a small-to-moderate effect.

**Figure 4:**
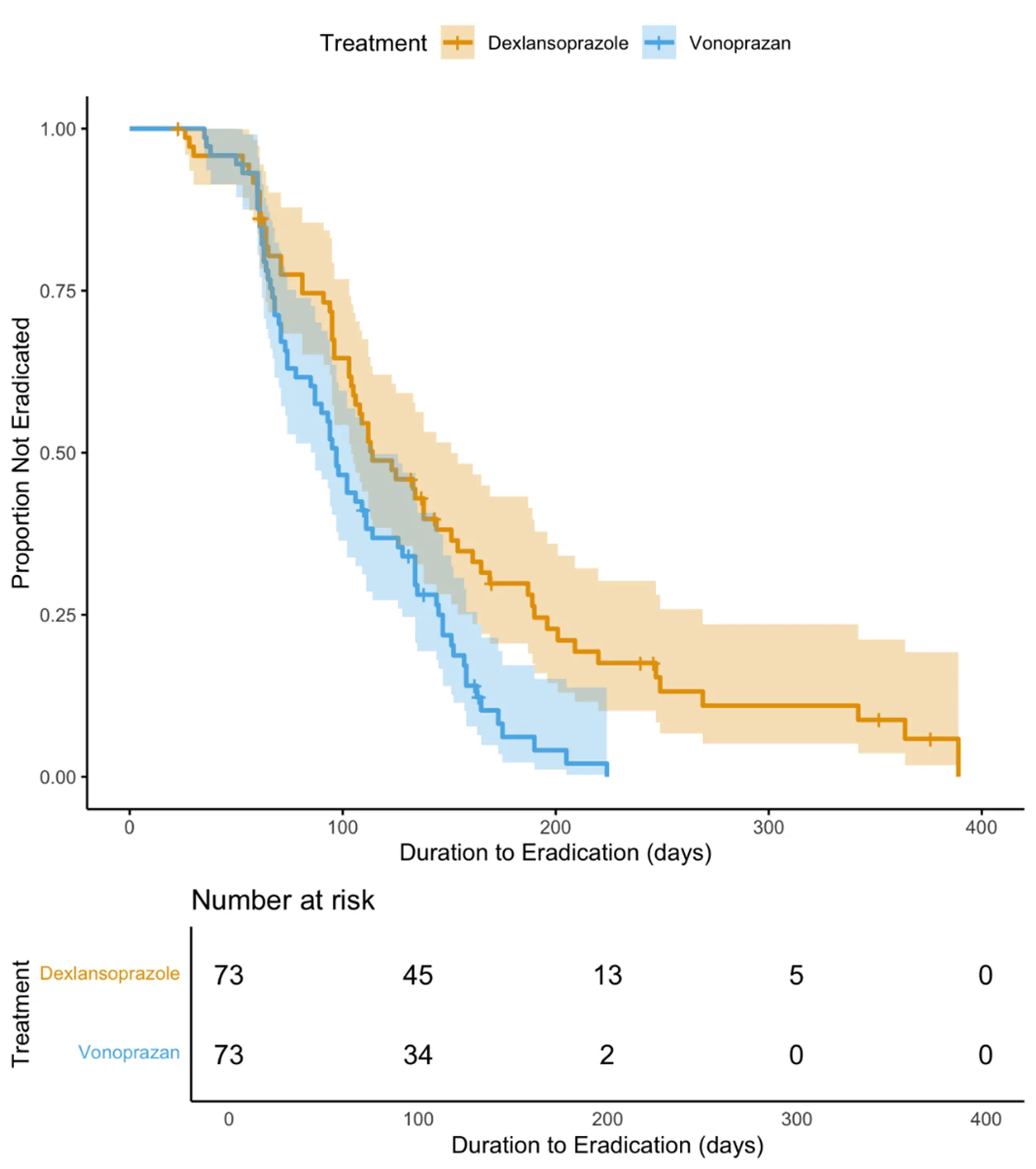
Kaplan-Meier curves comparing time to H. pylori eradication between vonoprazan and dexlansoprazole treatment groups For both treatment arms, n = 73. Time is shown in days from treatment completion to the date of the first documented negative urea breath test for each patient. Because confirmatory testing occurred at variable follow-up intervals in this retrospective cohort rather than at a single fixed time point, this duration differed across patients. Patients who did not achieve eradication or were lost to follow-up were censored at their last available observation. Statistical comparison between groups was performed using the log-rank test (p = 0.02). The number at risk at each time point is shown below the x-axis. H. pylori, Helicobacter pylori

Sensitivity analysis - standard antibiotic backbone

A sensitivity analysis restricted to the 144 patients (72 vonoprazan and 72 dexlansoprazole) who received the standard amoxicillin plus levofloxacin backbone (excluding two patients who received doxycycline substitution for quinolone allergy) confirmed the direction and magnitude of the treatment effect: vonoprazan-based therapy achieved eradication in 67 of 72 patients (93.06%) compared with 60 of 72 (83.33%) with dexlansoprazole-based therapy (risk difference +9.72 percentage points; χ² = 3.268, p = 0.07; Fisher exact p = 0.12). The virtually identical estimates confirm that the observed treatment effect is not driven by the small number of doxycycline substitutions.

Adverse events and tolerability

No patient discontinued treatment because of adverse events, although the retrospective design and absence of a structured symptom diary preclude reliable estimation of mild or transient adverse-event rates. No serious treatment-related adverse events were recorded in either arm.

## Discussion

In the eradication of *H. pylori*, PCABs have emerged as a promising alternative to PPIs, especially in cases requiring robust and sustained acid suppression. In our real-world comparative analysis, the vonoprazan-based regimen demonstrated a higher eradication rate than the dexlansoprazole-based regimen. Although the difference did not reach statistical significance at the conventional 95% CI, the consistent direction of effect across multiple measures suggests a potential clinical benefit. Point estimates of risk measures favored vonoprazan; however, the corresponding 95% CIs remained statistically inconclusive. Exploratory analysis at the 90% confidence level, used to identify potentially meaningful trends in underpowered studies, showed that the risk ratio and odds ratio excluded the null at this wider, non-confirmatory threshold, favoring vonoprazan; consistent with our Results, we interpret this as hypothesis-generating rather than as evidence of statistical superiority. The absolute risk difference of approximately 10 percentage points in the vonoprazan group is consistent with a potential clinical benefit, corresponding to a number needed to treat of 11. To address the uncertainty inherent in frequency-based analysis and further characterize the treatment effect, we conducted Bayesian analysis. The posterior distribution estimated a higher eradication rate with vonoprazan (93.3% vs. 83.6%) and a posterior risk difference of 9.3% (95% CrI: -1.1%, 20.1%). The posterior probability that the risk difference favored vonoprazan was 96.2%; however, because the credible interval still includes values close to and below zero, we regard this as a directional signal rather than confirmatory evidence of benefit. Kaplan-Meier analysis showed a numerically shorter median time to documented confirmation of eradication with vonoprazan (67 days) than with dexlansoprazole (80 days); because confirmatory testing intervals varied widely and were not protocol-driven in either arm, we present this as an exploratory finding rather than evidence that vonoprazan clears the infection biologically faster and we do not draw conclusions about symptom relief or complication risk from it.

Our directional findings are broadly in keeping with a larger body of real-world studies and clinical trials that have demonstrated superior eradication outcomes with vonoprazan compared with PPI-based therapies. A large cohort analysis reported higher eradication rates with vonoprazan triple therapy than with second-generation PPIs [22]. Similarly, another retrospective study found vonoprazan-based regimens more effective than conventional PPI-based therapies [23]. Meta-analyses also support these observations, showing significantly higher eradication success with vonoprazan in both first-line and rescue settings [24-26]. Randomized clinical trials from the United States, Europe, and Egypt further support vonoprazan's superiority across diverse populations [27,28]. Our findings are directionally consistent with these reports, with a 93.15% eradication rate observed with vonoprazan compared with 83.56% with dexlansoprazole. While our findings did not reach statistical significance, the observed effect size and direction of change support the growing evidence base favoring vonoprazan as a more potent acid suppressant in eradication regimens.

While our findings do not meet strict statistical thresholds at the 95% CI level, there is consistency in the direction of effect across multiple analyses. The absolute risk difference of approximately 10 percentage points observed in the vonoprazan group may, if confirmed in future studies, help reduce treatment failure and limit the need for retreatment or additional diagnostic testing. Adherence was assessed retrospectively through clinician interview and review of pharmacy dispensing records, and all patients included in the analysis met this composite adherence threshold; because this assessment relies on retrospective documentation rather than direct observation, it cannot fully exclude differential under-reporting of non-adherence between arms, and we note this as a constraint on, rather than a safeguard against, confounding by compliance. The observed advantage of vonoprazan over dexlansoprazole may stem from its CYP2C19-independent acid suppression, particularly relevant in Asian populations, where polymorphisms in this enzyme are common. Clinically, vonoprazan's rapid onset of action and stability in acidic environments are relevant pharmacological properties; because vonoprazan was administered at 20 mg twice daily in this study, while dexlansoprazole was the once-daily agent, dosing frequency does not represent an advantage of vonoprazan in this comparison. Whether the numerically higher and earlier documented eradication observed with vonoprazan translates into improved adherence, cost-effectiveness, quicker symptom relief, or a reduced risk of complications such as peptic ulcer recurrence was not assessed in this study, since we did not collect symptom, cost, quality-of-life, or complication data; we present these as hypotheses for future prospective studies rather than as findings supported by our data. Our findings may have implications for regional treatment guidelines, such as Maastricht VI and the Asian-Pacific consensus, which continue to evolve in response to emerging real-world data [21,29]. This study provides preliminary evidence that could inform future guideline updates, especially in populations with variable PPI response.

Limitations

This study has several limitations. First, the retrospective, single-center design may limit generalizability to other regions with different resistance profiles or health-system contexts. Second, treatment allocation was not randomized and was determined by physician preference; although demographic characteristics, diagnostic method, antibiotic backbone, and individual comorbidities (including diabetes, hypertension, hyperlipidemia, obesity, hypothyroidism, ischemic or cardiovascular disease, liver disease, vitamin B12 deficiency, anemia, and duodenal ulcer) were balanced between arms (Tables 1-2), structured data on prior antibiotic exposure, prior acid-suppressant use, and precise smoking pack-years were not consistently captured in the retrospective electronic medical record and could not be reconstructed on re-audit; residual confounding by these specific unmeasured factors cannot be excluded.

Third, the primary comparison did not reach conventional statistical significance at α = 0.05; the width of the 95% CIs around the treatment-effect estimates, rather than a post hoc power calculation, is the more informative indicator of the precision achieved at this sample size for a ~10 percentage-point difference. Fourth, susceptibility testing was not performed routinely, precluding adjustment for baseline resistance patterns; this is a genuine gap given levofloxacin resistance rates approaching one-third in Indian *H. pylori* isolates, since levofloxacin susceptibility, rather than metronidazole or clarithromycin, neither of which was used in this regimen, is the most directly relevant unmeasured factor here.

Fifth, the retrospective ascertainment of adverse events lacks the structure of a symptom diary and is likely to under-report mild reactions. Finally, while rapid urease test on biopsy is a well-validated diagnostic method, it does not permit inference about baseline antibiotic susceptibility of individual isolates. Adherence was assessed via a composite clinician-driven judgment rather than a structured pill-count or refill-log metric, which may not fully capture non-adherence. Larger, multicenter, prospective studies with adequate power to detect small-to-moderate treatment differences, routine antimicrobial susceptibility testing, and more complete capture of prior antibiotic and acid-suppressant exposure are warranted to confirm these findings.

## Conclusions

In this real-world retrospective comparative study, vonoprazan was associated with a numerically higher eradication rate and earlier documented confirmation of eradication compared with dexlansoprazole, along with favorable point estimates on exploratory risk measures; however, the primary comparison did not reach conventional statistical significance, and these findings should be interpreted as hypothesis-generating rather than as establishing superiority. Our findings also highlight the importance of evaluating clinical outcomes not only by statistical thresholds but also by their practical impact on patient care. Adherence in this study was assessed through a composite clinician-driven judgment based on follow-up interviews and pharmacy dispensing records, under which all patients met the adherence threshold. While no patient was excluded for non-adherence, this retrospective assessment cannot fully rule out differential under-reporting of non-adherence between arms.

To our knowledge, this is among the first real-world comparisons between vonoprazan and dexlansoprazole in India. These findings lay the groundwork for a larger, multicenter, prospective study to confirm or refute these observed differences across diverse populations. Furthermore, subgroup analyses exploring differential responses by age, sex, comorbidities, or antibiotic resistance profiles, integrated with real-world health economics, could provide a more nuanced understanding of treatment effectiveness and personalized treatment strategies.

## Appendices

Appendix

**Table 4:** Eradication success rates by treatment group PPI, proton pump inhibitor

Treatment group	Total patients (n)	Success (n)	Incidence risk (% with 95% CI)
Vonoprazan	73	68	93.2% (84.7% to 97.7%)
PPI	73	61	83.6% (73.1% to 91.2%)
Total	146	129	88.4% (82.0% to 93.1%)

**Table 5:** Bayesian estimates of treatment effectiveness for H. pylori eradication with vonoprazan vs. dexlansoprazole *H. pylori, Helicobacter pylori*; CrI, credible interval

Parameter	Value
Posterior success rate (Vonoprazan)	93.3% (95% CrI: 84.9%-97.0%)
Posterior success rate (Dexlansoprazole)	83.6% (95% CrI: 73.3%-90.3%)
Posterior absolute risk difference	9.3% (95% CrI: -1.1% to 20.1%)
Probability that vonoprazan is superior	96.2% (Risk difference > 0)
Prior distribution	Beta(1,1) non-informative prior
Sample size (each group)	73 patients
